# Supplementary material for: Are carbon emissions trading and green financial instruments synergistic? -Comprehensive quantitative research based on content analysis
Source: PLoS One. 2024 Mar 7;19(3):e0298601. doi: 10.1371/journal.pone.0298601 (PMC10919601; doi:10.1371/journal.pone.0298601)
Supplement: S1 Data — (ZIP) [file pone.0298601.s001.zip › Data/地级市Z_F工作报告.zip!/║╙─╧/╞╜╢Ñ╔╜╩╨/└┤╫╘╣1⁄2╓┌║┼í╛┬φ┐╦╩2╛▌═°í┐_2009.docx]

各位代表:

现在,我代表市人民政府,向大会报告政府工作,请予审议,并请各位政协委员提出意见。

一、过去五年工作回顾

本届政府任期的五年,是我市改革发展进程中极不平凡的五年。在省委、省政府和市委的正确领导下,在市人大、市政协的监督支持下,市政府团结带领全市人民,深入贯彻落实科学发展观,抢抓发展机遇,积极应对挑战,奋力开拓创新,圆满完成了市八届人大确定的各项目标任务,经济社会发展迈上新台阶。

——总体经济实力大幅提升。2008年全市生产总值1067.7亿元,是2003年的3倍,年均实际增长15.4%,总量由2003年的全省第7位上升到第4位;人均生产总值21998元,是2003年的3倍。地方财政一般预算收入62.5亿元、支出102.7亿元,分别是2003年的4.6倍和3.7倍,收入规模由全省第7位上升到第3位。

——农业、工业和服务业加快发展。粮食生产连续五年丰收,2008年总产量38.8亿斤,比2003年增产近10亿斤。全部工业增加值663.6亿元,第三产业增加值270亿元,分别是2003年的3.6倍和2.4倍;二、三产业比重由86.8%提高到90.5%。

——投资、消费和出口快速增长。2008年全社会固定资产投资421.1亿元,社会消费品零售总额245.8亿元,出口总额5.3亿美元,分别是2003年的5.4倍、2.2倍和5.4倍。

——人民生活水平显著提高。2008年城镇居民人均可支配收入13531元,农民人均纯收入4420元,分别是2003年的2倍和2.1倍。城乡居民储蓄存款余额540.3亿元,是2003年末的2.3倍。

——社会事业全面进步。教育、文化、卫生等事业蓬勃发展。食品药品安全监管体系逐步健全。科技进步贡献率明显提升。就业和社会保障工作不断加强。人口自然增长率控制在5.2‰。

五年来,我们所做的主要工作是:

(一)大力实施项目带动战略。在优化投资结构的基础上,强力推进项目建设,五年实施各类重点建设项目165个,带动全社会固定资产投资1363亿元,是上一个五年的4.7倍。兰南、宁洛、二广、郑尧高速公路平顶山段建成通车,总里程343公里,结束了我市没有高速公路的历史。燕山水库建设和昭平台水库除险加固工程顺利完成。6个220千伏、1个500千伏变电站建成投用,电网总变电容量达到508万千伏安。市污水处理厂二期和6县(市)污水处理厂全部建成,新增日污水处理能力20万吨。舞钢公司100万吨宽厚板生产线、姚电公司2×60万千瓦机组等66个工业项目建成投产,中平能化双30万吨氯碱暨45万吨电石、新平高工程等34个项目正加快建设。孟宝铁路复线、郑渝铁路客运专线等重大基础设施项目前期工作取得进展。大批项目的建成投产(投用),进一步夯实了发展基础,洗煤、焦炭、钢材、发电量、干法水泥、高压开关设备等主要产品产量实现翻番。

(二)积极引导产业结构优化。工业结构进一步改善。五年投入工业结构调整和高新技术产业化资金5.6亿元,优势产业和高新技术产业发展加快。2008年规模以上工业增加值突破500亿元,是2003年的4.9倍,其中高新技术产业比重21%,比2003年提高14.2个百分点。出台了促进30户重点工业企业加快发展的意见,引导新上项目向产业集聚区集中,企业规模和产业集中度明显提高,年销售收入超10亿元的企业由2003年的4家增加到14家。自主创新能力不断增强。确立了创新型平顶山建设目标,组织实施了特高压输变电装备、瓦斯抽采-发电-热害治理等重大科技专项。五年获国家技术发明二等奖1项、省科技进步奖63项,新建省级企业技术中心5家、省级企业工程技术研究中心7家、企业博士后科研工作站2家,新认定省级高新技术企业39家、高新技术产品45个,新增中国名牌产品4个、中国驰名商标1个。现代服务业发展明显提速。制定了加快服务业发展的政策措施,大力扶持文化旅游、现代物流、金融保险等现代服务业发展。2008年接待国内外游客720万人次,旅游总收入31亿元,分别是2003年的4.5倍和4.2倍;文化旅游产业占第三产业比重15.4%,比2003年提高7.6个百分点。各类物流企业47家,比2003年增加21家。金融服务领域不断拓宽,有效信贷投放大幅增加,有力支持了地方经济发展。2008年末金融机构各项存款余额812.8亿元、贷款余额442.8亿元,分别是2003年末的2.6倍和2.1倍。节能减排和环境保护成效显著。关闭小煤矿313个;淘汰小炼焦产能25万吨、立窑水泥产能500万吨、小火电机组46.2万千瓦;拆除黏土砖瓦窑厂581个,复垦整理土地2万亩,新型墙材应用比例达到85%。完成了各类燃煤发电机组脱硫设施改造。制定并实施了林业生态市建设规划,完成造林98.5万亩。与“十五”末相比,万元生产总值能耗下降13.2%,化学需氧量、二氧化硫排放量分别削减17.5%和10.4%,城区大气环境质量明显改善。

(三)扎实做好“三农”工作。强农惠农政策全面落实。2005年开始免征农业税,落实种粮直补、农资综合直补、良种补贴、农机具购置补贴等政策,农民由过去每年缴纳各类税费5亿元,变为现在获得政府补贴4亿元。现代农业加快发展。五年新增有效灌溉面积16万亩、农机总动力97万千瓦,新建标准化养殖小区366个、规模养殖场4800个,新增国家级农业产业化龙头企业1家、省级龙头企业9家、农民专业合作经济组织200余家。顺利完成了“农村百万劳动力转移工程”。新农村建设扎实推进。编制了市、县两级新农村经济发展规划和部分中心村建设规划,启动了联系中心乡(镇)、帮建中心村工作。所有行政村比省定目标提前一年实现通水泥路或油路,2007年实现户户通电。五年新解决30万农民的饮水安全问题,新建农村户用沼气13万座、便民超市2000个,23万农村人口实现脱贫。20户以上自然村广播电视“村村通”覆盖率达到95%以上。县域经济不断壮大。2008年6县(市)生产总值、财政一般预算收入占全市的比重分别达到64.9%和46.3%,比2003年提高1个和7.7个百分点;10个县(市、区)财政一般预算收入均超2.5亿元,最高的达9.1亿元。

(四)强力推进城镇建设和管理。城市功能日趋完善。深入开展城市创建活动,2006年成功创建中国优秀旅游城市之后,接着创建国家园林城市和国家卫生城市。老城区五年完成城建投资35亿元,新建、改扩建道路37条,新建桥梁16座,改造背街小巷65条,建成街头游园绿地106处。新城区累计完成投资86.8亿元,已建和在建的建筑面积达到423万平方米,各项配套设施正在完善,湖滨新城初具规模。城市绿化覆盖率、建成区绿地率分别达到36.2%和31.6%,比2003年提高6.4个和6.3个百分点;人均公共绿地面积8.1平方米,比2003年增加3.2平方米。县城和小城镇建设不断加强。各县(市)城市创建活动成效明显,修建、改建道路87条,建成各类广场、游园63个。城镇化率由2003年的31.4%提高到40.2%。城市管理水平进一步提高。大力推进市容环境、“城中村”、城区河道、集贸市场整治,城市面貌明显改观。

(五)不断深化改革开放。各项改革深入推进。全市国有企业改制面达94%,13家大中型企业全部实现股权多元化。平煤天安成功上市。中国平煤神马能源化工集团组建成立。56所企业学校全部移交市、县(区)管理。乡(镇)机构改革顺利完成。城市信用社组建为商业银行。城乡一体化和农村土地流转试点加快推进。粮食流通、集体林权、交通、供销、公用事业、收入分配等领域的改革取得新进展。开放水平不断提高。积极组团参加国家和省举办的重大招商引资和经贸洽谈活动,主动加强与发达地区的经济技术合作。2008年实际利用外资1.3亿美元,是2003年的4.7倍。文化、教育、科技等领域的对外交流与合作不断加强。非公有制经济快速发展。2008年实现增加值550亿元,是2003年的4倍,占生产总值的比重为51.5%,比2003年提高13.5个百分点。

(六)着力构建和谐社会。就业再就业工作成效显著。五年新增城镇就业45.8万人,其中下岗失业人员再就业18.5万人。动态消除了城镇“零就业家庭”。社会保障体系不断完善。建立健全了城镇职工养老、失业、医疗、工伤、女工生育保险制度,参保人数不断增加。城市低保巩固了动态管理下的应保尽保,人均月补差由55元提高到126元。在全省率先建立了农村低保制度。农村五保对象集中供养率由8%提高到42%。在全国率先创办了“爱心救助超市”。建立了新型农村合作医疗和城镇居民基本医疗保险制度,在全省率先实现了全民医保。五年新建经济适用房119万平方米,新增实物配租房1045套,发放住房公积金贷款11亿元。190万平方米的棚户区改造基本完工。社会事业加快发展。“两基”顺利通过国家验收。城乡免费义务教育全面实施。消除农村中小学D级危房37.7万平方米。在全省率先建立了高中阶段贫困生救助制度。高等院校办学水平进一步提升。完成了12所市、县医院和全部乡(镇)卫生院的改造,建成了7个疾病预防控制中心、81个社区卫生服务中心和2631个标准化村卫生所。在全省率先实施城市医院支持农村卫生工程。严格执行计划生育政策,稳定了低生育水平,人口计生工作连续14年受到省委、省政府表彰。残疾人事业健康发展,开始创建全国残疾人工作示范市。3项非物质文化遗产入选国家级保护名录。小品《高效率的爱》获中国曲艺最高奖——牡丹奖,豫剧《珠帘秀》、《李清照》获河南文华大奖,数字电影《女大学生部落》等10部作品获省“五个一工程”奖。市博物馆和市文化艺术中心主体完工。八集系列片《走遍中国·走进平顶山》在中央电视台播出,在海内外反响较好。群众体育运动蓬勃发展,业余训练水平不断提高。在北京奥运会和残奥会上,我市选手夺得3金1银1铜。精神文明和民主法制建设扎实推进。深入开展社会主义核心价值体系建设和精神文明创建活动,不断加强大学生和未成年人思想政治工作。五年新增全国文明单位和先进单位7个、省级文明单位183个。国防教育、双拥共建、人民防空工作深入开展,连续三届荣获全国双拥模范城称号。驻平部队和武警官兵在抗洪抢险、抗震救灾、维护稳定、支持地方建设等方面作出了重要贡献。法制宣传教育和依法治市工作取得显著成效。第五、第六届村委会换届选举顺利完成。安全生产和社会大局持续稳定。严格落实企业的安全生产主体责任和政府的监管责任,强化安全生产秩序整顿和隐患排查治理,保持了安全生产形势持续平稳。郏县高门垌煤矿事故抢险创造了我市煤矿救援史上的奇迹。积极开展食品药品安全专项整治,没有发生重大食品药品安全事件。加强社会治安综合治理,积极构建社会治安防控体系,深入开展“命案侦破攻坚战”和打黑除恶、打击“两抢一盗”等专项行动,社会治安形势明显好转。加强信访工作,解决了一批难点热点问题,维护了社会稳定。

(七)全面提升政府服务水平。自觉接受人大及其常委会的监督,支持政协参政议政,认真办理人大代表建议和政协提案。深入推进法治诚信服务型政府建设,加快推进以转变政府职能、转变工作作风、提高行政效能、提高公务员素质为重点的“两转两提”工作,政府服务水平明显提高。市级审批项目取消62%,行政审批效率位居全省前列。清理废止规范性文件487份。政府应急管理、市长热线、电子政务等工作不断加强。统计服务水平进一步提高。反腐倡廉工作取得新成效。审计、外事、侨务、对台、史志、物价、气象、烟草、盐业、民族宗教、新闻出版、国家安全等项工作都取得了新成绩。全市支援四川地震灾区工作扎实推进。

各位代表!

2008年,是我市近年来经济社会发展中遇到大事最多、挑战最大的一年。面对严峻的宏观经济形势,我们坚持咬定发展不放松,迎难而上、奋力拼搏,经济在复杂多变的环境中保持了平稳较快发展。生产总值比上年增长13.6%,地方财政一般预算收入增长24.8%,规模以上工业增加值增长19.8%,社会消费品零售总额增长23.5%,出口总额增长67.9%,城镇居民人均可支配收入、农民人均纯收入分别实际增长9.1%和11.2%,均高于全省平均水平,较好地完成了经济社会发展的主要目标任务,被省政府授予完成责任目标优秀单位称号。市委、市政府承诺的“十大实事”全部落实。成功举办了’2008华侨华人中原经济合作论坛、中国·宝丰第四届魔术文化节、第六届中国曲艺节和中原鲁山大佛开光等重大节会活动,充分展示了平顶山形象。

各位代表!

过去的五年,是我市积极实践科学发展观的五年,是发展速度明显加快、发展质量不断提高、发展后劲显著增强的五年,是民生持续改善、人民群众得实惠更多的五年,是城乡面貌变化巨大、平顶山知名度和影响力快速提升的五年。五年的拼搏奋斗,为进一步发展奠定了坚实基础;五年的探索实践,为今后工作积累了宝贵经验。我们深切体会到,做好政府工作,必须以解放思想为先导,创新发展思路。要始终坚持“学先进、比创新、看实效”,坚决破除不合时宜的旧观念、旧体制,勇于探索符合实际的新思路、新举措,加快建设全面发展的新型现代化城市、中原城市群重要的战略支点和全国重要、国际知名的旅游目的地。必须以项目建设为龙头,加快发展步伐。要始终坚持把项目建设作为加快经济社会发展的重要抓手,坚定不移地谋划好项目、建设大项目,既拉动当前经济增长,又为今后发展备足后劲。必须以节能减排为突破口,建设生态文明。要始终坚持绿色发展理念,强力推进节能减排和林业生态建设,破解资源环境瓶颈制约,加快构建资源节约型和环境友好型社会。必须以引进战略投资者为重点,推进改革开放。要始终坚持用开放的思维和改革的办法解决前进中的困难和问题,大力推进体制机制创新,着力加强与国内外大企业、大集团的战略合作,不断优化资源配置,增强产业竞争力。必须以改善民生为着力点,促进社会和谐。要始终坚持以人为本,高度重视保障和改善民生,把更多的财力、更大的精力向基层和困难群体倾斜,让人民群众共享改革发展成果。必须坚持依法行政,改进政府工作。要始终坚持按照法定权限和程序行使权力、履行职责,推进阳光政务,提高行政效能,努力建设人民满意的政府。

各位代表!

这些成绩的取得,是省委、省政府和市委正确领导的结果,是市人大、市政协积极支持的结果,是全市人民共同奋斗的结果。在此,我代表市人民政府,向工作在全市各条战线的广大工人、农民、知识分子、干部、驻平部队指战员、武警官兵和公安民警致以崇高的敬意!向关心、支持我市现代化建设的各界人士和国际友人、港澳台同胞表示衷心的感谢!

在肯定成绩的同时,我们也清醒地看到,政府工作与人民的期望还有差距,经济社会发展中还存在一些不容忽视的矛盾和问题。

一是主要指标按人均计算还处于全省中游水平，农民人均纯收入还比较靠后。二是服务业发展相对滞后，第三产业比重低于全省平均水平。三是节能减排任务艰巨，万元生产总值能耗高于全省平均水平。四是一些关系人民群众切身利益的问题解决得还不够好，部分低收入群众生活比较困难。五是安全生产基础仍然薄弱，部分行业和领域安全事故时有发生。六是少数干部作风不实，行政效率不高。当前，受国内外经济形势的影响，部分企业生产经营困难，经济下行和财政保障压力加大，大学生和返乡农民工就业困难增加。对此，我们一定高度重视，采取有效措施，认真加以解决，绝不辜负人民的期望和重托！

二、今后五年奋斗目标

今后五年，是我市全面建设小康社会的关键五年。我们要在国际金融危机影响日益加深的形势下谋求又好又快发展，要在中原城市群你追我赶的竞相发展中保持走在全省前列的良好态势，挑战和压力前所未有。但大挑战蕴藏着大机遇，大压力孕育着大动力。应该看到，我市拥有丰富的自然资源、文化资源和较好的产业基础，区位条件、要素成本优势在提升，又处于工业化、城镇化快速推进时期，经济发展的潜力巨大。更重要的是，我们仍处于重要战略机遇期，经济发展的基本面和长期向好的态势没有改变；国家和省出台的一系列应对挑战、促进增长的政策措施，有利于我们争取更多支持，解决自身难以解决的问题；全市广大干部群众坚持“学先进、比创新、看实效”，干事创业的氛围更加浓厚。这些都是我们应对严峻复杂形势、推进经济社会进一步发展的难得机遇和力量源泉。只要我们坚定信心、抢抓机遇，就一定能够克服困难、化“危”为“机”。

今后五年经济社会发展的奋斗目标是:继续保持走在全省前列的良好态势，基本实现全面建设小康社会的目标。主要有三个标志:一是力争实现“两个翻番”。到2013年，全市生产总值、地方财政一般预算收入均比2008年翻一番，总量分别突破2100亿元和120亿元。人均生产总值超过5800美元，基本达到全面小康标准。二是力争实现“两个基本完成”。第一产业增加值比重降到8%以下，从事二、三产业的劳动力比重接近60%，基本完成工业化；城镇人口达到250万人，城镇化率接近50%，基本完成城镇化。三是力争实现“两个明显高于”。生产总值、财政一般预算收入、城乡居民收入等主要经济指标增速明显高于全省平均水平，科研经费支出占生产总值比重、居民平均受教育年限、千人拥有医生数、社会保障覆盖率等社会事业发展指标明显高于全省平均水平。

围绕上述目标，必须在科学发展观的指引下，加快构建“一个载体、三个体系”。“一个载体”，就是建设产业集聚区，通过产业的集聚促进生产要素的规模集聚和优化组合，形成新的竞争优势和发展动力，进而带动社会就业，用产业和就业创造需求，用需求再来拉动产业发展，促进城镇化与工业化互动融合，打造资金、技术和人才高地。“三个体系”，一是现代产业体系，加快构建现代农业、工业主导产业、高新技术产业、现代服务业和基础产业相互支撑、互动发展的产业体系，着力打造战略支撑产业，加快工业化进程；二是现代城镇体系，按照中原城市群发展规划，着力构建中心城市、县城、中心镇、中心村协调发展的城乡布局结构，以城镇基础设施的集中建设，引导产业的集聚发展，实现资源的集约利用，促进人口向城镇集中，加快城镇化进程；三是自主创新体系，重点抓好一批重大科技专项，建设一批工程研发中心和重点实验室，建立健全产学研有效结合的科技成果转化体系，为率先实现崛起提供科技支撑。

实现上述目标，要做到“五个着力”:一是着力扩大社会需求。紧紧抓住国家扩内需的有利时机，千方百计抓好项目建设，增加固定资产投资；积极扩大消费需求特别是居民消费需求，增强消费对经济增长的拉动作用。同时，要努力保持外贸稳定增长。二是着力转变发展方式。切实加强自主创新，加快形成以科技进步与创新为基础的创新体系；大力发展现代服务业，加快形成三次产业协调发展的新格局；积极推进生态文明建设，努力实现又好又快发展。三是着力推进改革开放。加大重点领域和关键环节的改革力度，大力实施开放带动主战略，进一步壮大非公有制经济，加快形成科学发展的体制机制，建设更加开放的平顶山。四是着力优化发展环境。进一步深化行政管理体制改革，提高政府行政效能和服务水平，使行政审批效率整体位居全省前列，努力创造更加宽松的发展环境。五是着力构建和谐社会。积极扩大社会就业，进一步提高城乡居民收入，大力发展社会事业，不断完善社会保障体系，扎实推进平安建设，确保社会更加和谐稳定。

我们相信，经过五年的努力，平顶山的经济将更加繁荣、社会将更加和谐、城乡将更加美好、人民将更加幸福，一个更加大气、更加和气、更富朝气、充满活力的新鹰城，必将焕发出更加绚丽的光彩！

三、2009年工作任务

2009年是新一届政府团结带领全市人民应对危机、共克时艰、抢抓机遇、谱写美好生活新篇章的起始之年。政府工作的总体要求是：以邓小平理论和“三个代表”重要思想为指导，深入贯彻落实科学发展观，紧紧围绕保持经济平稳较快发展、保持走在全省前列的良好态势这一首要任务，坚持“学先进、比创新、看实效”，着力扩内需、保增长，调结构、上水平，抓改革、增活力，重民生、促和谐，全面推进社会主义经济建设、政治建设、文化建设、社会建设和生态文明建设，努力实现经济社会发展新跨越。经济社会发展的主要预期目标是:生产总值、地方财政一般预算收入均增长12%，力争增长15%；全社会固定资产投资增长20%；社会消费品零售总额增长15%；外贸出口增长10%，实际利用外资增长13%；居民消费价格涨幅4%左右；城镇居民人均可支配收入、农民人均纯收入均增长8%；城镇新增就业6.2万人，城镇登记失业率控制在4.5%以内；人口自然增长率控制在6.5‰以内；万元生产总值能耗降低4.8%，化学需氧量、二氧化硫排放量分别削减2.4%和8.2%。筹措资金34.4亿元，办好关系人民群众切身利益的“十大实事”。

重点抓好八项工作:

(一)全力保持经济平稳较快发展。坚持统筹兼顾、突出重点、狠抓关键，着力保持经济平稳较快发展的良好势头。

努力扩大投资需求。坚持争取政策支持和吸引社会投资两手抓，大力实施“8185”投资促进工程，在能源化工、装备制造、农林水利、交通运输、城镇建设、产业升级、节能减排、社会事业等8大领域，安排100个重点项目，总投资规模800亿元，确保今年完成全社会固定资产投资500亿元以上。工业投资完成280亿元以上。开工建设华能郏县煤矸石综合利用发电、平顶山南500千伏超高压输变电工程等19个项目；建成中平能化集团双30万吨氯碱、舞钢公司新宽厚板生产线调整等19个项目；加快推进鲁阳发电公司一期2×100万千瓦机组、海星化工30万吨煤焦油等14个续建项目建设；切实做好舞钢公司特宽厚板轧机易地改造、鲁阳发电公司二期2×100万千瓦机组等项目前期工作。交通投资完成60亿元以上。开工建设汝登高速平顶山段、孟宝铁路复线等项目；建成焦柳线平顶山段电气化改造、范辛铁路平顶山段、长安大道西段延伸等项目；积极推进叶舞高速公路建设；加快环城快速通道、郑渝铁路客运专线平顶山段、鲁山军用机场增建民用工程等项目前期工作；积极争取平顶山至郑州城际轨道交通项目。农林水利投资完成60亿元以上。开工建设南水北调中线工程平顶山段、市农产品质量安全监测中心、市“12316”农业信息服务系统等项目；完成已开工建设的19座、争取再开工建设13座中小型水库除险加固工程；加快孤石滩灌区续建配套和节水改造工程；切实做好北汝河治理前期工作。城市基础设施和社会事业投资完成100亿元以上。开工建设市垃圾处理工程等项目；建成市区污水处理厂一期工程技改、新城区污水处理厂一期、矿区采煤沉陷区治理、市疾控中心综合实验楼等项目；加快推进河南质量工程职业学院国家食品安全检测科研中心建设；积极争取国家级产品质量监督检验中心项目。加大工作力度，及时解决问题，创造良好环境，确保重点项目早开工、早投产、早见效。

积极扩大消费需求。努力提高消费能力。推进事业单位工资制度改革，落实企业最低工资制度，健全农民工工资支付保障机制。继续提高企业退休人员基本养老金水平，人均月增100元以上。提高农村五保对象集中供养标准，由每人每年1600元提高到2000元。提高优抚对象、建国前老党员等抚恤和生活补助标准。继续落实对农民的各项补贴政策，扩大补贴范围，提高补贴标准。落实好大中型水库移民后期扶持等政策。积极培育消费热点。引导房地产企业多开发建设中小套型、中低价位普通商品房，鼓励有实力的房地产企业集中连片开发，促进房地产市场稳定健康发展。大力发展社区商业、物业、家政等便民消费，加快发展旅游休闲消费，扩大文化娱乐、体育健身等服务消费。继续优化消费环境。落实居民购买住房、汽车等大宗消费的鼓励政策，全面清理消费领域相关规费，积极发展消费信贷。继续实施“万村千乡市场工程”和“新网工程”，新建260家农村连锁超市和一批配送中心。做好“家电下乡”、“农机下乡”、“汽车、摩托车下乡”等工作。加强“菜篮子”工程建设，抓好现有农贸市场的提档升级，在市内三区各扩建或再建一个大中型农贸综合市场。大力整顿和规范市场秩序，维护消费者合法权益。

着力保持外贸稳定增长。落实鼓励出口的各项政策，加大对中平能化集团、舞钢公司等重点出口企业和高压开关、电收尘器、碳化硅等重点出口产品的扶持力度，支持农产品出口。鼓励紧缺资源、先进技术和关键设备进口。

切实加强财政金融和主要生产要素保障。认真落实财政金融政策。努力扩大政府投资，引导带动社会投资。依法加强税收征管，规范非税收入管理。优化支出结构，确保工资发放、法定支出、规定配套、“十大实事”等资金需要。鼓励金融机构扩大信贷投放，改进金融服务，更好地支持地方经济发展。加强与省金融机构的战略合作，抓紧落实已签约的千亿元授信资金。积极引进外地股份制银行在我市设立分支机构。支持中小金融机构拓展业务范围，加大对重点企业、重点项目、中小企业、“三农”、城市基础设施建设的投入力度。实施重点上市后备企业培育工程，支持企业上市融资。加强金融生态环境建设，完善企业和个人征信体系。支持保险业改革和发展。着力提高土地保障能力。坚持最严格的耕地保护制度和最严格的节约用地制度，统筹安排各类用地，优先保障重点企业、重点项目需求。积极开展土地整治，力争盘活存量土地2000亩以上。大力推进多层标准厂房建设和用地方式改革。做好第二次土地调查工作。完成市、县、乡土地利用总体规划修编。主动帮助企业排忧解难。认真开展“企业服务年”活动，实行市级领导联系百户重点企业制度，帮助企业解决实际困难和问题。全面落实各项涉企优惠政策，切实减轻企业负担。继续组织银行、企业、项目单位开展银企洽谈、供需对接等多种活动，促使银企之间、企业之间形成稳定的合作、协作关系。加强经济运行调控，保障重点企业生产要素供给。

(二)加快推进经济结构战略性调整。坚持经济结构适应性调整和战略性调整相结合，推动发展方式转变。

加快构建现代产业体系。着力推动工业支柱产业优化升级。围绕能源化工、装备制造、特宽厚钢、新型建材、农产品加工等优势产业做大做强，积极推广信息技术和信息产品，加快应用先进适用技术改造提升传统产业。大力推进中平能化集团1万吨锦纶66中低旦工业丝、平煤机液压支架控制系统、中材环保袋式除尘产业化等重大产业升级项目建设。着力推动30户重点企业发展，尽快培育一批大型企业集团。大力发展高新技术产业。研究制定鼓励高新技术产业发展的政策措施，以电子信息、机电一体化、新材料、新能源和生物技术等为重点，培育壮大高新技术产业。重点抓好中平能化集团对位芳纶产业化、蓝彩新型平板显示器、新平高特高压超高压开关重大装备产业基地等项目建设。申报高新技术企业5家以上。加快发展服务业。设立服务业发展引导资金，重点支持文化旅游、现代物流等现代服务业发展。围绕优势旅游资源和丰厚的文化底蕴，高标准编制旅游产业发展规划，突出抓好尧山、香山寺、佛泉寺、风穴寺、叶县县署、二郎山、三苏园等精品景区建设，积极开发特色旅游产品。加大宣传推介力度，拓展旅游市场，提升服务水平。加快编制物流产业发展规划，以平东、平西火车站为依托，积极推进一批物流项目建设。

加快构建现代城镇体系。着力加快中心城区建设步伐。全力推进国家园林城市、国家卫生城市创建工作，确保今年“创园”成功、明年“创卫”成功。做好迎接中国优秀旅游城市复检工作。老城区改造要突出道路交通、“城中村”改造和园林绿化三个重点。进一步完善城市道路网络，开工建设香山路、凌云路南延等15个道路桥梁项目，新增城市道路10公里以上。成立专门机构，引进有实力的房地产开发公司，强力推进郝堂、大营等15个“城中村”的改造。搞好香山公园、生态文化体育公园等6个绿化项目建设，建成区绿化覆盖率达到37%以上。抓好第四水厂续建和城区西水东调工程建设。新建一批环卫设施。新城区建设要完善基础设施和服务功能，新增道路通车里程8公里、建筑面积25万平方米、绿化面积20万平方米，建成区面积达到13平方公里。加快新城区医院、商场、公交枢纽站等配套设施建设，提升承载能力，加快聚集人气。努力提高城市管理水平。强力推进城市环卫体制改革。严格落实创建工作制度，大力推进市场、“城中村”、餐饮业和“五小”门店、背街小巷、城乡接合部的综合整治，逐步取缔马路市场。加强道路、桥梁、路灯、雨污水管网等市政设施养护。统筹推进城乡一体化发展。加强土地利用总体规划、城乡规划、产业集聚区规划的统筹、协调和衔接。完成平宝叶鲁一体化规划、中心城区50平方公里控制性详细规划、市域村镇体系规划的编制工作。加强县城和小城镇建设，每个县(市)再有2个小城镇基础设施建设达标。优化公共资源配置，引导人口、产业、生产要素向城镇集中，力争全市城镇化率达到42%以上。

加快构建自主创新体系。以企业为主体，以重点项目为依托，以技术和资本为纽带，努力推动企业与高校、科研机构开展多种形式的合作，增强核心竞争力。大力实施人才强市战略，加快引进经济社会发展急需的高层次人才和智力。支持企业建立研发中心，申报省级工程技术研究中心5家、省级企业技术中心3家。推动产业关键技术研发及产业化，启动实施2项重大科技专项。大力实施名牌战略和标准化战略，新增省级以上名牌产品4个、省优质产品5个、采标确认产品3个。加强农作物良种培育和产业化开发。做好知识产权创造、应用和保护工作。

加快发展产业集聚区。抓住我市化工产业集聚区、高新技术产业集聚区等10个产业集聚区列入全省重点支持范围的机遇，全面加快区内规划和基础设施建设，提升承载能力，推动企业向园区集中、园区向城镇发展、劳动力向城镇转移，努力把产业集聚区建成科学发展的载体。结合产业集聚区规划建设，优化县域产业布局，引导城市农产品和矿产品加工企业向原料产地转移，促进县域产业集约、集群发展，提升县域经济发展水平。

(三)全面发展农业和农村经济。认真落实今年中央1号文件精神，大幅度增加对农村基础设施和社会事业的投入，大幅度提高政府土地出让收益、耕地占用税新增收入用于农业的比例，大幅度增加对农村公益性建设项目的投入，促进农村稳定发展、农民持续增收。大力发展农业产业化经营。稳定发展粮食生产，切实抓好叶县、郏县高产稳固区和汝州低产改造区建设，确保全市粮食种植面积稳定在610万亩以上，力争再夺丰收。大力发展高效经济作物和特色农业，重点抓好油料、烟叶等大宗经济作物和食用菌、名贵中药材等特色农产品生产。进一步落实促进畜牧业发展的各项政策，加快发展规模化养殖和畜产品加工业，加强重大动物疫病防控和畜产品质量安全管理。积极推进农业标准化生产，推行农产品市场准入制度。实施农业产业化龙头企业“培育工程”，新增省级龙头企业3家以上。规范发展农民专业合作经济组织100家以上。扎实推进新农村建设。加快编制和实施新农村经济发展规划和新村建设规划，上半年完成539个中心村的规划编制。加大对中心乡(镇)、中心村的支持力度，筹措1亿元，对按照中心村建设规划建房的农户给予每户3000元建筑材料的引导性补贴。加快基础设施建设，新建、改建农村公路1000公里；解决15万人的饮水安全问题；全面完成20户以上自然村广播电视“村村通”工程；新建户用沼气3.5万座、大型工程沼气10处。加大对贫困地区特别是革命老区建设的支持力度，完成98个贫困村的整村推进扶贫，解决和巩固6万贫困人口的温饱问题。做好南水北调丹江口库区移民安置工作。深入推进农村各项改革。认真落实推进土地承包经营权流转的政策措施，建立土地流转有形市场和信息发布平台，新增土地流转面积10万亩以上。充分发挥涉农金融机构作用，改善和创新农村金融服务。加快推进村镇银行建设步伐。深化农村综合改革，提高农业公共服务能力。

(四)继续加大改革开放力度。深入推进各项改革。加快商贸物资流通企业改制步伐，基本完成已进入程序的国企改制任务。积极推进市属企业与中央、省管企业及非公企业之间的战略合作。支持企业兼并重组、强强联合，鼓励关联产业、上下游企业一体化经营。支持农村信用社组建农村商业银行。大力推进财税、投资、价格体制改革，深化医药卫生体制改革，积极稳妥地推进事业单位分类改革，加快城市供水、污水处理企业产权制度改革和接待单位改革。进一步扩大对外开放。加大招商选资力度，精心组织参加第四届中博会、第十三届中国国际投洽会等重点招商活动，突出办好鹰城文化宝岛行、第二届“华合论坛”等重大经贸活动，办好第三届世界叶氏联谊会。对引进项目，要全面考虑利税、就业、环保等各方面因素，既算经济账，又算社会账、政治账；既算当前账，又算长远账、综合账。继续实施“走出去”战略，鼓励优势企业到境外投资办厂，带动设备、技术及劳务输出。大力发展非公有制经济。发挥中小企业发展专项资金引导作用，着力扶持100户重点非公企业做大做强。开展“百名企业家培训工程”。完善中小企业信用担保体系及担保风险补偿机制，支持民营企业组建融资担保公司，努力解决融资难问题。

(五)切实加强文化建设。大力繁荣文化事业。深入推进文化精品工程和“五个一工程”，做好《墨子》等电影的制作发行工作。组织好第二届中国戏剧奖·梅花表演奖大赛、首届中国曲剧文化艺术节和第三届中国书法“兰亭奖”大赛等活动。加强文化基础设施建设，市博物馆、市文化艺术中心建成投用，扩建改造30个乡(镇)综合文化站，建成3个文化信息资源共享工程县级支中心、916个村级基层站点和300个农家书屋。筹建山顶公园广播电视发射塔。继续实施农村电影放映工程，开展“舞台艺术送农民”活动。做好重点文物和非物质文化遗产保护和开发利用工作。加强互联网等新兴媒体建设与管理。加快发展文化产业。着力培育龙头演艺团体，积极引导宝丰民间演艺业快速发展。科学制定汝瓷发展规划，全面加快汝瓷产业发展。以绢花、泥塑、五谷画、金镶玉为龙头，带动根雕、奇石、剪纸、烙画等工艺美术市场趋向繁荣。积极发展汝陶、汝帖、叶县编钟、舞钢龙泉剑、应国青铜器等高知名度文物的复仿制业。切实搞好文化产业园区的规划建设。着力打造文化品牌。加大文化资源包装、开发、推介力度，努力将香山寺风景区、佛泉寺风景区、汉唐皇家温泉打造成具有世界吸引力的文化旅游品牌。围绕香山观音文化，创作一台高水平的舞台剧，打造国家舞台艺术精品。积极挖掘开发牛郎织女文化。策划拍摄电视连续剧《叶公》，推出一批历史文化名人系列剧目。继续深化文化体制改革。加快推进文化市场综合执法改革。继续深化公益性文化事业单位改革。稳妥推进专业文艺表演团体改革和经营性文化事业单位转企改制。加快建设宝丰文化改革发展试验区。拓宽文化产业融资渠道，鼓励民间资本进入文艺表演、文化娱乐、出版物分销等领域。切实加强精神文明建设。认真开展学习实践科学发展观活动，提高干部群众推动科学发展的能力和水平。以社会主义核心价值体系教育为重点，深入开展“知荣辱、讲正气、促和谐”主题道德实践活动。做好大学生和未成年人思想政治工作。大力开展优美环境、优良秩序、优质服务和创建文明单位、文明社区、文明景区竞赛活动，继续推进科教、文体、法律、卫生进社区和文化、科技、卫生“三下乡”活动，提高城乡文明程度。

(六)更加注重以改善民生为重点的社会建设。以办好“十大实事”为重点，着力解决关系人民群众切身利益的问题，切实把发展成果体现在人民群众幸福指数的提升上。

千方百计扩大就业。研究出台全民创业实施意见，以创业促就业。认真落实促进高校毕业生就业的各项政策措施，鼓励大学生到基层、中小企业和非公企业就业或自主创业。继续延长“4050”人员享受公益性岗位就业的各项补贴政策，动态消除城镇“零就业家庭”。支持困难企业采取在岗培训、轮班工作、协商减薪等办法，尽量不裁员或少裁员。继续做好复转军人就业安置工作。落实支持农民工返乡创业的政策措施，促进农民工就业。继续实施“农村劳动力技能就业计划”、“阳光工程”和“雨露计划”，完成农民职业技能培训6.5万人，新增转移就业5.3万人。

进一步完善社会保障体系。继续做实企业职工基本养老保险个人账户。启动农村养老保险试点工作。完善失业保险制度。全面实施城镇居民基本医疗保险制度。加强新型农村合作医疗工作，筹资标准提高到100元。强力推进高危高风险行业和领域职工参加工伤保险。完善城乡低保制度，做到应保尽保。进一步办好“爱心救助超市”。大力发展社会福利事业和慈善事业。开工建设经济适用房20万平方米、竣工12万平方米；开工建设廉租房3万平方米、竣工1.6万平方米；发放租赁补贴1100万元、住房公积金贷款2.3亿元；完成棚户区改造配套工程；改造农村贫困残疾人危房300户，努力实现住有所居的目标。

大力发展教育、卫生等社会事业。坚持优先发展教育。落实好城乡免费义务教育政策。提高农村义务教育公用经费标准。改造10所农村薄弱初中。选派100名市直学校教师到农村对口支教。认真解决农民工子女在流入地免费接受义务教育问题。积极化解农村“普九”债务。大力推进职业教育攻坚计划，创建1~2所省级示范性职业院校，建设2个职业教育专业实训基地；进一步整合教育资源，依托河南城建学院、平顶山学院两个老校区，组建两所规模均为5000人以上、各具特色的中等职业学校；加快发展农村职业教育并逐步实行免费。提高高等教育质量，促进高等院校办出特色、办出水平。鼓励和规范民办教育发展，支持民办学校引进优质教育资源联合办学。认真落实资助家庭经济困难学生的政策措施。加快发展医疗卫生事业。扩大免费公共卫生服务范围，增加重大传染病、慢性病和职业病防治专项投入。为白内障患者全部免费实施复明手术，实现全国白内障无障碍市创建目标。完成市中医院门诊楼建设任务，为3所县医院、65所乡(镇)卫生院配备医疗设备，对30所乡(镇)卫生院进行提档改造。继续实施城市医院支援农村卫生工程。广泛开展爱国卫生运动。做好人口和计划生育工作。严格实行“一票否决”制，稳定低生育水平。实施出生人口缺陷干预，进一步提高出生人口素质。加强流动人口计划生育服务管理。综合治理出生人口性别比偏高问题。将农村部分计划生育家庭奖励扶助标准由人均600元提高到900元。积极创建全国人口与计划生育综合改革示范市。进一步加强老龄工作。广泛开展全民健身活动，继续实施农民体育健身工程。

强化安全生产和食品药品安全监管。积极开展“质量和安全年”活动，建立有效的激励机制和监督约束机制，加强全员、全过程、全方位质量和安全管理。继续做好中小煤矿复产验收、停产整改和关闭退出工作，切实打好瓦斯治理、水害防治和技术改造三个攻坚战，坚决遏制重特大事故发生。加大安全投入，着力抓好煤矿、交通运输、非煤矿山、消防、危险化学品、民爆器材和公共聚集场所等重点行业和领域的安全专项整治，保持安全生产形势持续平稳。贯彻落实《食品安全法》，积极创建食品安全优秀城市和食品安全示范县(市、区)，深入开展食品药品安全和食品添加剂专项整治，让人民群众买得放心、吃得安心、用得舒心。

全力维护社会和谐稳定。加强信访工作，引导群众以理性合法的形式表达利益诉求，及时依法合理解决群众反映的问题。强化源头治理，有效防范和妥善处置企业破产、征地拆迁、农村土地纠纷等可能引发的群体性事件。做好法制宣传教育、人民调解和法律援助工作。扎实开展平安创建活动，加强社会治安综合治理，严密防范、严厉打击各类违法犯罪，增强人民群众的安全感。支持工会、共青团、妇联等人民团体充分发挥桥梁纽带作用和参与社会事务管理的作用。完善村民自治，加强村务公开、民主管理。加强社区建设，完善服务功能，提高服务水平。深入开展国防教育和动员，支持驻平解放军和武警部队建设，不断提高民兵预备役部队和人民防空建设质量；积极开展双拥共建活动，巩固和发展军政军民团结。同时，继续做好外事、侨务、对台、史志、地质、气象、民族宗教、国家安全、防震减灾和对口支援江油恢复重建等工作。

(七)加快推进生态文明建设。严格实行问责制和“一票否决”制，确保完成节能减排目标，为经济社会发展腾出环境容量和承载空间。大力推进节能降耗。重点抓好工业、交通、建筑等关键领域的节能工作。严把新建项目准入关，从源头控制高耗能、高污染项目发展。抓好一批重点节能工程，着力支持舞钢公司余热余压综合利用、巨龙淀粉锅炉改造等项目建设。建立重点耗能企业能源利用状况网上监测平台，对年综合能耗5千吨标准煤以上的企业实行动态监管。加强节能管理，党政机关、公共企事业单位要发挥表率作用。强力推进污染减排。完成第一次污染源普查工作。巩固和深化燃煤锅炉整治成果，完成35吨以上燃煤锅炉烟气脱硫除尘工程。启动中盐皓龙平顶山分公司搬迁工作。加大水污染防治力度，污水处理平均负荷率达到85%以上。加强对饮用水源地的保护。做好农村畜禽养殖企业污染减排和治理。通过努力，出境水质达标率达到70%以上，城市集中饮用水源地水质达标率100%，城区大气环境质量优良天数保持在300天以上。积极发展循环经济。抓好煤矸石、粉煤灰的综合利用，加强瓦斯抽采、尾矿水利用和再生资源回收等工作，提高资源利用率。编制和实施新型墙材产业发展规划，推动新型墙材产业做大做强，产能达到45亿标块，工业固体废弃物综合利用率达到75%以上。加快林业生态建设。深化集体林权制度改革，调动全社会育林护林的积极性。抓好山区生态林业体系、农田防护林体系、生态廊道网络、城郊和村镇绿化等重点工程，完成造林27.5万亩。

(八)进一步加强政府自身改革与建设。紧紧围绕保增长、保民生、保稳定这个大局，扎实推进“两转两提”，为经济社会发展创造良好环境。

加快转变政府职能。积极稳妥地推进政府机构改革，上半年完成市级政府机构改革，年底前完成县级政府机构改革。深化行政审批制度改革，进一步减少和规范行政审批事项。推进部门行政审批职能向审批服务科集中、审批服务科向审批服务大厅集中，各单位进厅项目要到位、对科室的授权要到位，确保所有审批事项的承诺时限达到全省第一。加快推进行政事业性收费改革，停止和取消行政事业性收费100项。健全政府绩效考核体系，引导各方面工作科学发展、和谐发展。进一步扩大政府信息公开，增强政府工作透明度和公信力。完善应急预案体系和管理体制，有效应对自然灾害、事故灾难、公共卫生和社会安全突发事件。

大力推进依法行政。严格依照法定权限和程序履行职责、接受监督。严格执行人大及其常委会的决议、决定，自觉接受人大及其常委会的法律监督和工作监督；加强与人民政协的协商，自觉接受人民政协的民主监督；广泛听取各民主党派、工商联和无党派人士的意见和建议。认真办理人大代表建议和政协提案。落实重大行政决策听取意见、合法性审查、集体决定、责任追究等制度，提高决策规范化、制度化水平。搞好第二次经济普查，为科学决策提供依据。建立健全规范性文件发布、备案和定期清理制度。继续推行行政执法责任制，规范行政处罚裁量权。加强行政复议，化解行政争议。重视发挥新闻舆论和社会公众的监督作用。

进一步转变工作作风。牢固树立为基层服务、为企业服务、为群众服务的意识，深入调查研究，及时发现问题、解决问题。坚持求真务实，力戒官僚主义、形式主义，切实做到“严、细、深、实、快”。创新公务员激励约束机制，搞好公务员培训教育，开展争做“人民满意的公务员”活动，强化行政效能监察，努力建设一支政治坚定、业务精通、清正廉洁、作风优良的公务员队伍。

切实加强廉政建设。深入开展反腐倡廉教育，加强制度建设，从源头上预防腐败。严格执行领导干部廉洁自律规定，清清白白做人，干干净净做事。树立过紧日子的观念，对公务购车用车、会议经费、公务接待费用和出国出境经费等支出实行负增长。切实加大审计监督力度，加大案件查处力度，对各种违法违纪行为严惩不贷。我们一定勤勉尽责，以实际行动和工作业绩，建设为民、务实、廉洁、高效的政府，让人民放心，让人民满意。

各位代表！

雄关漫道真如铁，而今迈步从头越。在过去的岁月中，勤劳智慧的鹰城人民已经创造出了辉煌的业绩，站在了新的历史起点上。在今后的征程上，尽管还有不少困难和曲折，但是，我们坚信：前途是光明的，任何艰难险阻也挡不住英雄的平顶山人民阔步前进的步伐！让我们在省委、省政府和市委的领导下，团结起来，同心同德，克难攻坚，扎实工作，朝着全面建设小康社会的宏伟目标奋勇前进！我们的目的一定要达到，我们的目的一定能够达到
